# Antiplatelet efficacy of ticagrelor versus clopidogrel in Mediterranean patients with diabetes mellitus and chronic coronary syndromes: A crossover pharmacodynamic investigation

**DOI:** 10.3389/fcvm.2022.1057331

**Published:** 2022-11-22

**Authors:** Ana Lucrecia Marcano, Montserrat Gracida, Gerard Roura, Josep Gomez-Lara, Rafael Romaguera, Luis Teruel, Lara Fuentes, Guillem Muntané-Carol, Oona Meroño, Silvia Gabriela Sosa, Joan Antoni Gómez-Hospital, Josep Comin-Colet, José Luis Ferreiro

**Affiliations:** ^1^Department of Cardiology, Bellvitge University Hospital, L’Hospitalet de Llobregat, Barcelona, Spain; ^2^Bio-Heart Cardiovascular Diseases Research Group, Bellvitge Biomedical Research Institute (IDIBELL), L’Hospitalet de Llobregat, Barcelona, Spain; ^3^Faculty of Medicine, University of Vic-Central University of Catalonia, Barcelona, Spain; ^4^Department of Cardiology, Bellvitge University Hospital, Centro de Investigación Biomédica en Red de Enfermedades Cardiovasculares, L’Hospitalet de Llobregat, Barcelona, Spain

**Keywords:** ticagrelor, chronic coronary syndrome, antiplatelet therapy, high platelet reactivity, diabetes mellitus

## Abstract

**Introduction:**

Patients with diabetes mellitus (DM) have augmented platelet reactivity and diminished responsiveness to clopidogrel. Ticagrelor, a more potent P2Y_12_ inhibitor, is clinically superior to clopidogrel in acute coronary syndromes, although its role in chronic coronary syndromes (CCS) is still the subject of debate. The aim of this investigation was to compare the pharmacodynamic effectiveness of ticagrelor and clopidogrel in Mediterranean DM patients with CCS.

**Materials and methods:**

In this prospective, randomized, crossover study, patients (*n* = 20) were randomized (1:1) to receive, on top of aspirin therapy, either ticagrelor 180 mg loading dose (LD)/90 mg maintenance dose (MD) b.i.d. or clopidogrel 600 mg LD/75 mg MD o.d. for 1 week in a crossover fashion with a 2–4 week washout period between regimens. Platelet function measurements were performed at 4 timepoints in each period (baseline, 2 h and 24 h after LD, and 1 week), including light transmission aggregometry (LTA, primary endpoint), VASP assay, Multiplate and VerifyNow P2Y_12_.

**Results:**

The ticagrelor LD achieved greater platelet inhibitory effect than clopidogrel LD, assessed with LTA (20 μM ADP as agonist), at 2 h (34.9 ± 3.9% vs. 63.6 ± 3.9%; *p* < 0.001) and 24 h (39.4 ± 3.5% vs. 52.3 ± 3.8%; *p* = 0.014). After 1 week of therapy, platelet reactivity was again significantly inferior with ticagrelor compared to clopidogrel (30.7 ± 3.0% vs. 54.3 ± 3.0%; *p* < 0.001). The results were consistent with the other platelet function assays employed.

**Conclusion:**

In Mediterranean patients with DM and CCS, ticagrelor provides a more potent antiplatelet effect than clopidogrel after the LD and during the maintenance phase of therapy.

**Clinical trial registration:**

[ClinicalTrials.gov], identifier [NCT02457130].

## Introduction

Subjects with diabetes mellitus (DM) have a higher risk of developing cardiovascular disease and experiencing atherothrombotic events, which have poorer prognosis than those occurring in patients without DM (1). One of the factors involved in the augmented atherothrombotic risk of DM patients with coronary artery disease (CAD) is a hyper-reactive platelet phenotype, which contributes to an impaired responsiveness to antiplatelet drugs, mainly to clopidogrel (2, 3). Therefore, the augmented ischemic risk among DM patients with CAD clearly emphasizes the need to optimize platelet inhibition in this population with the goal of ameliorating clinical outcomes (4).

The use of more potent and less variable P2Y_12_ receptor antagonists such as prasugrel or ticagrelor has demonstrated a reduction in adverse ischemic events when compared to clopidogrel in patients suffering an acute coronary syndrome (ACS) (5, 6). However, the observed clinical superiority of ticagrelor or prasugrel over clopidogrel in ACS patients has not been replicated in patients with stable CAD or undergoing elective percutaneous coronary intervention (PCI) (7, 8). In fact, clopidogrel is still widely used in real-life clinical practice as part of dual antiplatelet therapy (DAPT), e.g., in patients undergoing elective PCI or in those with stabilized symptoms after an ACS following a strategy of DAPT de-escalation. It is well established that clopidogrel has a large interindividual variability in response with genetic factors, such as polymorphisms of cytochrome P450 (CYP) isoforms (mainly CYP2C19), playing a key role in this phenomenon (9, 10). Evidently, the prevalence of genetic polymorphisms may vary greatly among races and, therefore, it is relevant that pharmacodynamic (PD) investigations take into consideration ethnicity when evaluating antiplatelet agents.

Since the evidence regarding the PD effectiveness of clopidogrel compared to ticagrelor in DM patients with a chronic coronary syndrome (CCS) is relatively scarce (11, 12), we designed the Comparison of Ticagrelor and clopidogrel in patients with Coronary artery disease and type 2 Diabetes Mellitus (TICS-DM) study, with the aim of assessing the platelet inhibitory effects of these two P2Y_12_ inhibitors in a Mediterranean population with a comprehensive panel of platelet function assays.

## Materials and methods

### Subject population and study design

This was a prospective, open-label, two-sequence, two-period, randomized, crossover study conducted in Mediterranean (Spanish nationality) type 2 DM patients with 18–75 years of age and known stable CAD (angiographically documented) on a background of aspirin therapy (NCT 02457130). The World Health Organization criteria were used to define DM status. Exclusion criteria included: known allergies to clopidogrel or ticagrelor, blood dyscrasia or bleeding diathesis, any recent acute coronary event (<1 year), hemodynamic instability, recent treatment with any other antiplatelet agent (<14 days) with the exception of aspirin, oral anticoagulation with a coumarin derivative, any active bleeding or malignancy, history of stroke (<6 months prior to inclusion) or any intracranial bleeding, platelet count <100 × 10^6^/μl, severe chronic kidney disease (creatinine clearance <30 ml/min) and pregnant females.

**FIGURE 1:**
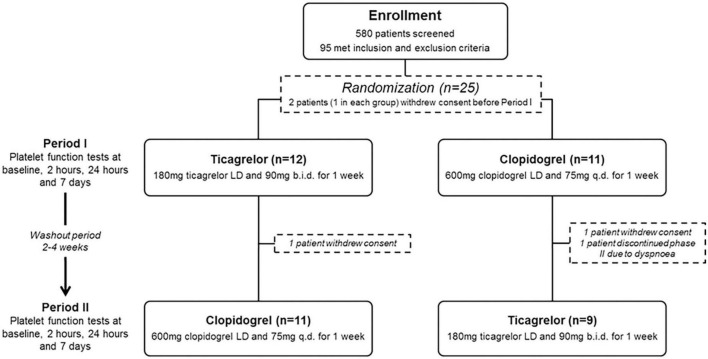
Flow diagram of the study design and enrollment process. LD, loading dose.

Subjects were randomized in a 1:1 fashion to ticagrelor [180-mg loading dose (LD) followed by 90-mg maintenance dose] or clopidogrel (600-mg loading dose followed by 75-mg daily maintenance dose) for 1 week (Figure 1). All patients were on chronic aspirin therapy (100 mg o.d.), that was maintained at the same dose throughout the study. Patients crossed-over treatment regimen after a 2 to 4-week washout period. Blood sampling for platelet function measurements were performed at the two phases of the study at the following timepoints: (1) baseline, (2) 2 h after LD, (3) 24 h after LD, and (4) 7 days (in the morning, with last dose of study drug administered the previous day). The washout periods were included in order to minimize carryover effects. A follow-up visit was performed at least 2 weeks after the last dose of the study drug to verify the absence of adverse events. Patient compliance was assessed by pill counting and interview.

The study was performed in compliance with the Declaration of Helsinki and was approved by the institutional Ethics Committee. All subjects included provided written informed consent.

### Sample collection and platelet function assays

Blood samples for platelet function assessment were collected at the scheduled time points from an antecubital vein; the first 2–4 ml of blood were discarded in order to avoid spontaneous platelet activation. Samples were processed by trained laboratory personnel (blinded to allocated treatment). Platelet function tests (PFT) included light transmission aggregometry (LTA), flow cytometric analysis of the phosphorylation status of the vasodilator-stimulated phosphoprotein (VASP), multiple electrode aggregometry (MEA) and VerifyNow P2Y_12_ (VN-P2Y_12_) assay.

#### Light transmission aggregometry

**FIGURE 2:**
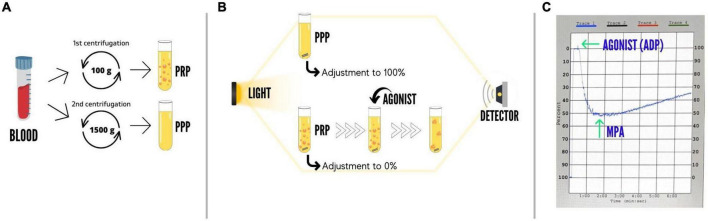
Schematic representation of the light transmission aggregometry assay. **(A)** Sample preparation. Platelet-rich plasma (PRP) is obtained as a supernatant after centrifugation of citrated blood at 100 *g* for 10 min; afterward, platelet-poor plasma (PPP) is obtained by a second centrifugation of the blood fraction at 1500 *g* for 15 min. **(B)** Methodology. In the aggregometer, light transmission is adjusted to 0% for PRP and to 100% for PPP for each measurement; during light transmission aggregometry, samples are constantly stirred at 1000 rpm; the addition of an agonist (ADP, arachidonic acid, collagen, etc.) to the PRP causes platelet aggregation, which is reflected by an increase in light transmission; platelet aggregation is monitored for at least 6 min. **(C)** Example of an aggregation curve. Baseline tracings are observed for stability and oscillations before the addition of an agonist (e.g., ADP); which can be seen in the curve as a spike; results are commonly reported as maximal platelet aggregation, which represents the maximal amplitude or% aggregation during the monitoring period. ADP, adenosine diphosphate; MPA, maximal platelet aggregation; PPP, platelet-poor plasma; PRP, platelet-rich plasma.

Light transmission aggregometry (a schematic example is shown in Figure 2) was performed according to standard protocols (13). Briefly, platelet aggregation was assessed using platelet-rich plasma (PRP) and platelet-poor plasma (PPP) by the turbidometric method in a two-channel aggregometer (Chrono-Log 490 Model, Chrono-Log Corp., Havertown, PA, USA). PRP was obtained as a supernatant after centrifugation of citrated blood at 100 *g* for 10 min and PPP was obtained by a second centrifugation of the blood fraction at 1500 *g* for 15 min. Light transmission was adjusted to 0% for PRP and to 100% for PPP for each measurement. Maximal platelet aggregation (MPA) was stimulated by 20 and 5 μmol/L adenosine diphosphate (ADP) as agonists. High on-treatment platelet reactivity (HPR) was defined as MPA > 64.5% and >42.9% with ADP 20 and 5 μmol/L, respectively (14).

#### Vasodilator-stimulated phosphoprotein assay

Vasodilator-stimulated phosphoprotein-phosphorylation (VASP-P) is a marker of the P2Y_12_ receptor reactivity and, therefore, P2Y_12_ inhibitors-induced inhibition. VASP was assessed according to standard protocols (15). Adding ADP to PGE_1_-stimulated platelets diminishes PGE_1_-induced VASP-P levels. If P2Y_12_ receptors are successfully inhibited, the addition of ADP will not decrease the PGE_1_-stimulated VASP-P levels. VASP-P levels were quantified with labeled monoclonal antibodies by flow cytometry with the Platelet VASP-FCM kit (Biocytex Inc., Marseille, France). The platelet reactivity index (PRI) was calculated once measured the VASP-P levels after stimulation with PGE_1_ (MFI PGE_1_) and also PGE_1_ + ADP (MFI PGE_1_ + ADP) with the following formula: PRI = ([MFI PGE_1_]–[MFI PGE_1_ + ADP]/[MFI PGE_1_]) × 100%. A reduced PRI indicates a greater inhibition of the P2Y_12_ signaling pathway, and a cut-off point of ≥50% PRI was utilized to define low responsiveness (16).

#### Multiple electrode aggregometry

Multiple electrode aggregometry (MEA) was assessed in whole blood with the Multiplate analyzer (Roche Diagnostics, Basel, Switzerland), which measures the change in impedance caused by platelets adhesion onto silver-covered electrodes working as sensor units (17). Curves were recorded for 6 min and platelet aggregation was determined as area under the curve of arbitrary aggregation units (AU*min) using 6.4 μmol/L ADP as agonist. The cut-off value used to define HPR was >468 AU*min (16).

#### VerifyNow P2Y_12_ assay

The VerifyNow System is a turbidimetric based optical detection system which measures platelet induced aggregation as an increase in light transmittance (Accumetrics, San Diego, CA, USA) and was utilized according to manufacturer’s instructions (18). The VerifyNow P2Y_12_ Assay measures changes in platelet function specific to P2Y_12_ inhibition by combining ADP + PGE_1_ stimuli. The reagents are incorporated into the assay channel to induce platelet activation and light transmittance increases as activated platelets bind and aggregate fibrinogen-coated beads. The device then measures this change in optical signal and reports results in P2Y_12_ Reaction Units (PRU). A cut-off point of >208 PRUs was used to define HPR (16).

### Study endpoints and sample size calculation

The primary endpoint of the present study was the comparison of MPA measured with LTA (20 μM ADP as agonist) and achieved after 1 week of therapy with ticagrelor or clopidogrel using the treatment regimens described above. An initial sample size of 30 patients was planned, but a mid-course recalculation of the sample size due to an overestimation of the standard deviation was performed and specified in an amendment to the protocol. The revised calculation of the sample size was as follows: assuming a standard deviation of MPA of 13 (19, 20), a difference between treatment groups of 10 with 90% power and 2-sided alpha = 0.05 will be detected with 18 completed subjects per regimen group. Randomization of a total of 25 subjects was allowed, considering an approximate dropout of 25%, in order to ensure that complete data from 18 subjects would be available for analysis.

Other secondary end points included: (a) evaluation of platelet reactivity between clopidogrel and ticagrelor with all the PFT after 1 week of treatment; (b) comparison of the 2 treatment regimens at 2 and 24 h after LD with all the PFT; and (c) determination at the different time points assessed of the proportion of patients with HPR (measured with all tests).

### Statistical analysis

Baseline continuous variables are expressed as mean ± SD, while categorical variables are reported as frequencies and percentages. Only those subjects who successfully completed the two treatment periods were considered for analysis. All statistical comparisons of platelet reactivity for the primary and secondary endpoints were performed using linear mixed-effects models with treatment, sequence, period, and treatment*period (treatment by period interaction to test for carryover effects) as fixed effects, subject as a random effect, and the baseline value of each corresponding platelet function test (MPA, PRI, AU*min, or PRU) as a covariate. Results are reported as least-squares mean (LSM) ± standard error of the mean (SEM). Comparisons between HPR rates were conducted using the McNemar test or the binomial exact test. All the analyses performed were evaluated with a 2-tailed probability value <0.05 to indicate a statistically significant difference. Statistical analysis was performed using SPSS version 18.0 software (SPSS Inc., Chicago, IL, USA).

## Results

Among 580 patients screened for eligibility, 95 met inclusion and exclusion criteria. Of these, 25 patients agreed to participate and were randomized to start with ticagrelor (*n* = 13) or clopidogrel (*n* = 12). Following randomization, four patients withdrew consent and one patient discontinued ticagrelor treatment due to side effects (dyspnea). Therefore, 20 patients successfully completed the two periods of the study and were included in the analysis. The flow chart of the study is illustrated in Figure 1, whereas baseline demographics and clinical variables are reported in Table 1. No significant dissimilarities were found between patients that initiated with either ticagrelor or clopidogrel. Among patients that completed the two phases of the study, 4 (20%) developed mild and transient dyspnea on ticagrelor therapy whereas no patient on clopidogrel therapy developed dyspnea. No patient experienced any ischemic or bleeding event during the study.

**TABLE 1:** Baseline characteristics.

	*n* = 20
Age, mean ± SD	65.45 ± 4.88
Male gender, n (%)	16 (80)
BMI, median [IQR]	29.7 [27.4–32.5]
**Cardiovascular risk factors**	
Active smoking, n (%)	1 (5)
Hypertension, n (%)	16 (80)
Dyslipidemia, n (%)	18 (90)
Peripheral artery disease, n (%)	3 (15)
Chronic kidney disease, n (%)	2 (10)
Prior stroke, n (%)	0
DM complications*, n (%)	8 (40)
Insulin treatment, n (%)	7 (35)
Oral antidiabetics, n (%)	20 (100)
**Cardiovascular history**	
Prior myocardial infarction, n (%)	14 (70)
Diseased vessels, mean ± SD	2.15 ± 0.75
Prior PCI, n (%)	17 (85)
Prior CABG, n (%)	4 (20)
LVEF, mean ± SD	58.5 ± 9.0
**Laboratory measurements**	
HbA1c, median [IQR]	6.8 [6.4–7.9]
Hb, mean ± SD	13.62 ± 1.66
Platelet count (×10^3^), mean ± SD	228 ± 51
MPV, mean ± SD	11.45 ± 1.10

*Complications of DM: Neuropathy, nephropathy, retinopathy, or vasculopathy. BMI. body mass index; CABG, coronary artery bypass grafting; DM, diabetes mellitus; LVEF, left ventricular ejection fraction; MPV, mean platelet volume; PCI, percutaneous coronary intervention.

### Pharmacodynamic effects of ticagrelor vs. clopidogrel

**FIGURE 3:**
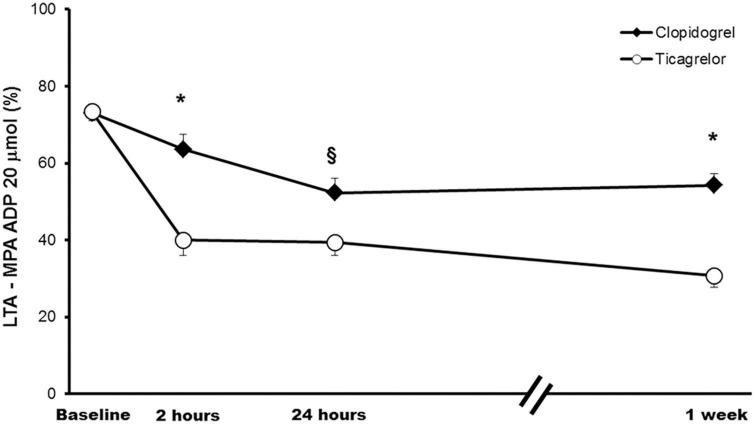
Platelet reactivity across study time points. Comparison of platelet reactivity over time measured with LTA and using 20 μmol ADP as agonists (primary endpoint). Values are expressed as least-squares means. Error bars indicate standard errors of the mean. *p < 0.001; ^§^ *p* < 0.05. ADP, adenosine diphosphate; LTA, Light transmission aggregometry, MPA, maximal platelet aggregation.

**FIGURE 4:**
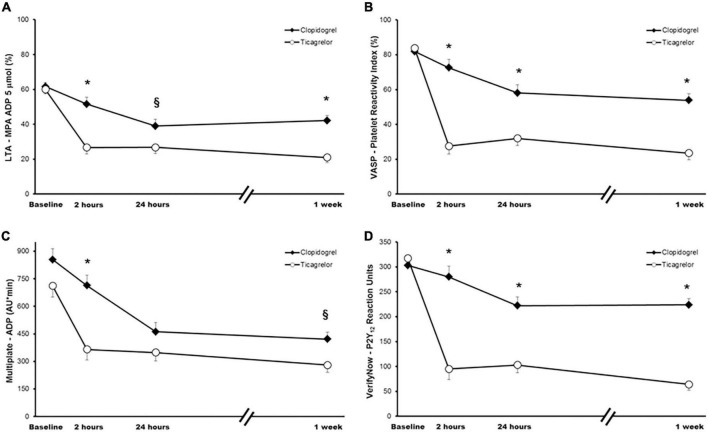
Platelet function measurements across study time points. **(A)** Light transmittance aggregometry using 5 μM adenosine diphosphate (ADP) as agonist. **(B)** Flow cytometric VASP analysis. **(C)** Multiple electrode aggregometry using ADP as agonist. **(D)** VerifyNow P2Y_12_ assay. Values are expressed as least-squares means. Error bars indicate standard errors of the mean. *p < 0.001; ^§^*p* < 0.05. LTA, light transmission aggregometry; MPA, maximal platelet aggregation; VASP, vasodilator-stimulated phosphoprotein.

At baseline, there were no statistical differences between the two regimens studied. After 1 week of treatment, MPA (using 20 μM ADP as agonist, the primary endpoint of the present investigation) was significantly lower (Figure 3) with ticagrelor compared to clopidogrel (MPA: 30.7 ± 3.0% vs. 54.3 ± 3.0%; *p* < 0.001). When assessing the PD efficacy of the LD, ticagrelor also provided greater platelet inhibition than clopidogrel both at 2 h (MPA: 34.9 ± 3.9% vs. 63.6 ± 3.9; *p* < 0.001) and 24 h (MPA: 39.4 ± 3.5% vs. 52.3 ± 3.8%; *p* = 0.014), as shown in Figure 3. No statistically significant differences were found by sequence, period, or the treatment-by-period interaction, which suggest no carryover effect. Similar findings were observed with 5 μM ADP and the other platelet function tests employed, showing greater inhibition of platelet aggregation at 2 h, 24 h, and 1 week in the ticagrelor group compared with the clopidogrel group (Figure 4). Of note, no differences in clopidogrel- or ticagrelor- mediated platelet inhibition were found when comparing patients with or without insulin therapy (data not shown).

### High platelet reactivity rates according to treatment

**FIGURE 5:**
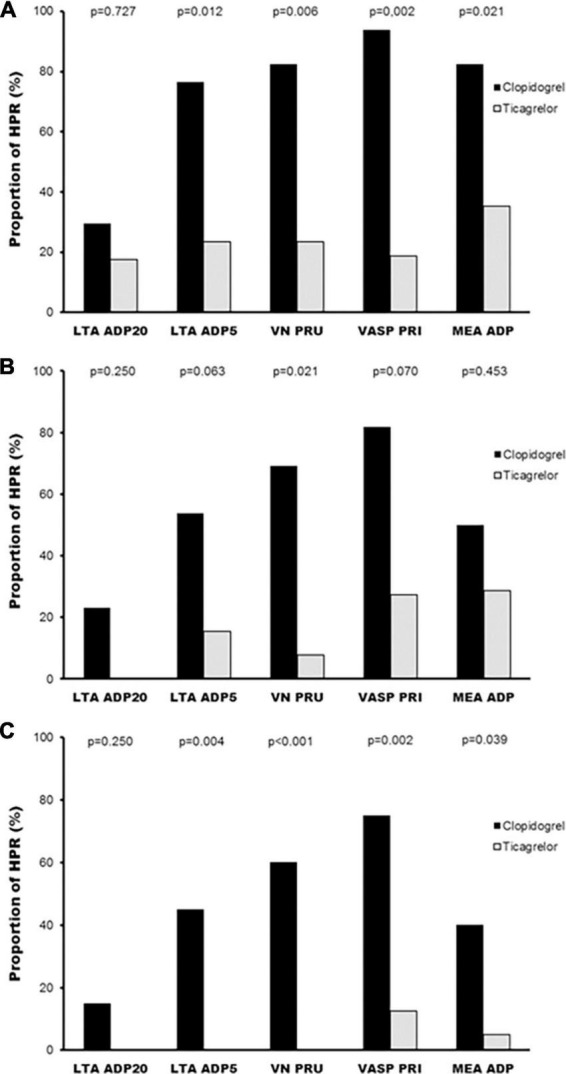
Percentage of patients with high platelet reactivity according to treatment with all platelet function tests and agonists. **(A)** HPR rates at 2 h; **(B)** HPR rates at 24 h; **(C)** HPR rates at 1 week. HPR, high on-treatment platelet reactivity; LTA, light transmission aggregometry; MEA, multiple electrode aggregometry; PRI, platelet reactivity index; PRU, P2Y_12_ reaction units; VN, VerifyNow; VASP, vasodilator-stimulated phosphoprotein.

Ticagrelor HPR rates ranged from 17.6 to 35.3% at 2 h, from 0 to 28.6% at 24 h, and from 0 to 12.5% at 1 week depending on the platelet function assay employed, whereas HPR rates with clopidogrel were higher, ranging from 29.4 to 93.8% at 2 h, from 23.1 to 81.8% at 24 h, and from 15.0 to 75.0% at 1 week, reaching statistical significance in most of the comparisons (Figure 5).

## Discussion

The present study was specifically designed to compare the antiplatelet effect of ticagrelor and clopidogrel in Mediterranean patients with DM and CCS, consisting on stable patients with prior ACS or coronary revascularization. The main finding of this investigation is that in such patients the PD benefit of ticagrelor over clopidogrel is maintained. Indeed, a LD of ticagrelor 180 mg has a faster and greater effect on platelet inhibition compared to the LD of clopidogrel 600 mg, an effect that is seen as soon as 2 h after intake of the LD of the drug. These outcomes were also consistently observed during the maintenance phase of therapy. This PD effect translated into ticagrelor achieving significantly lower rates of HPR at any time point of the study and with all platelet function tests employed.

Compelling data from previous PD investigations have demonstrated a greater, and also faster, inhibition of platelet reactivity achieved with ticagrelor compared with clopidogrel (19, 21). However, very few studies have addressed this issue in DM patients, a subpopulation at high risk of recurrent ischemic events. Of note, the available studies addressing this issue are actually *post-hoc* analyses and, thus, are not exclusively performed in DM patients (11, 22). In addition, it is quite relevant to consider ethnicity when evaluating responsiveness to antiplatelet agents (11, 23). In fact, the prevalence of loss-of-function alleles of the CYP2C19 isoform varies greatly among races (9), which has a huge impact on clopidogrel responsiveness. This investigation is, to the best of our knowledge, the first to specifically compare the antiplatelet efficacy of ticagrelor vs. clopidogrel in a Mediterranean Caucasian population with DM and provides a valid confirmation of the PD superiority of ticagrelor over clopidogrel irrespective of ethnicity.

DM patients have augmented platelet reactivity, leading to greater rates of HPR to clopidogrel than non-DM subjects, which is clearly associated with poorer clinical outcomes (2–4). This problem has incited the evaluation of more potent antiplatelet regimens in this high-risk population. The PD effectiveness in DM patients of other P2Y_12_ inhibition strategies, more potent than clopidogrel, has been compared among them in a number of mechanistic studies. For instance, in the CLOTILDIA study, ticagrelor displayed a greater platelet inhibitory effect than high-dose clopidogrel (150 mg daily) in stable patients at least 1 month after PCI (12). More importantly, a number of PD investigations have compared the platelet inhibitory efficacy of ticagrelor vs. prasugrel specifically in DM patients (24–26), although results were not completely consistent. Briefly, two studies have suggested separately a slightly greater antiplatelet efficacy of ticagrelor, although no differences in the rates of HPR to both agents were observed in any of these studies (24, 25). However, in the comprehensive OPTIMUS-4 investigation, the platelet inhibitory effectiveness of both agents were similar with most of the platelet function assays employed to evaluate the LD and MD regimens (26). In line with these findings, Galli et al. (27) observed in a recent investigation a similar PD efficacy of ticagrelor and prasugrel, after switching from clopidogrel, both in patients with and without DM; of note, despite an important increase in platelet inhibition after escalation of antiplatelet agents, platelet reactivity persisted higher among DM patients compared to those without DM.

Whether there is a clinical advantage of one of the two more potent P2Y_12_ antagonists, prasugrel, or ticagrelor, in DM patients is yet to be determined. In fact, in a prespecified analysis of patients with DM of the ISAR-REACT 5 trial, conducted in ACS patients with planned invasive therapy, no differences in ischemic or hemorrhagic events were seen between prasugrel and ticagrelor (28). The latter is in contrast with the somewhat surprising findings of the main trial, in which prasugrel significantly reduced the rates of the primary efficacy outcome, a composite of death, myocardial infarction and stroke (29, 30).

The favorable PD profile of ticagrelor in CAD patients with DM may contribute to explain the consistent benefit in terms of reduction of atherothrombotic outcomes observed in large-scale clinical trials that have evaluated different antiplatelet regimens with ticagrelor in several scenarios across the CAD spectrum. In the DM subgroup of the pivotal PLATO trial, dual antiplatelet therapy (DAPT) with ticagrelor diminished ischemic events compared to DAPT with clopidogrel in ACS patients at moderate to high ischemic risk, without differences in major bleedings (31). Nevertheless, the relative benefit achieved with ticagrelor in DM patients, although consistent with the global trial results, was somewhat attenuated (17 vs. 12% relative risk reduction of ischemic events in non-DM and DM patients, respectively), since a numerical (although not statistically significant) reduction of the occurrence of the primary efficacy endpoint was observed. In a different clinical setting, the addition of ticagrelor on top of aspirin as secondary prevention in patients with a prior myocardial infarction, which was evaluated in the PEGASUS-TIMI 54 trial, led to a significant reduction of recurring ischemic events with ticagrelor (pooled doses of 60 and 90 mg b.i.d.) compared to the control arm (aspirin monotherapy), including both cardiovascular and coronary heart disease mortality in the DM subgroup, although with the counterpart of a heightened risk of major bleeding (32, 33). Interestingly, a platelet function substudy of this trial showed a similar platelet inhibition of ticagrelor 60 mg and 90 mg b.i.d. doses regardless of diabetes status (34). More recently, the THEMIS trial, conducted in stable DM patients with CAD and without a history of myocardial infarction or stroke, showed that adding ticagrelor to aspirin resulted in a reduction of ischemic cardiovascular events albeit at the cost of a higher rate of major bleedings, when compared to aspirin monotherapy (35). Overall, these findings underline the need for carefully addressing the ischemic and bleeding risk of each and every patient in order to decide the most suitable antiplatelet strategy.

Clopidogrel is the preferred P2Y_12_ antagonist in patients with stable CAD undergoing PCI but it is also commonly prescribed in ACS patients deemed not suitable for potent DAPT due to increased bleeding risk. Moreover, the results of recent trials have suggested that a de-escalation of dual antiplatelet therapy (DAPT) strategy by reducing the intensity of DAPT through switching from more potent P2Y_12_ inhibitors (i.e., prasugrel or ticagrelor) to clopidogrel, could be useful to reduce hemorrhagic events in ACS patients at high risk of bleeding without losing efficacy in terms of preventing ischemic events (36–38). For these reasons among others, clopidogrel is still widely utilized in real-life clinical practice as part of DAPT (39, 40). However, the superior platelet inhibitory effect of prasugrel or ticagrelor compared to clopidogrel, as shown in the present study and other abovementioned investigations, suggest that high-risk subgroups such as DM patients may obtain a greater benefit from maintaining more potent antiplatelet regimens. Noteworthy, recent evidence points toward a potential benefit of personalized antiplatelet therapy using platelet function of genetic assessment (e.g., guided escalation of P2Y_12_ inhibitors) in the PCI setting, which may be of particular relevance in DM patients due to the heightened platelet reactivity and the high rates of clopidogrel suboptimal response that characterize this population (41, 42). Indeed, an individualized approach taking into consideration the balance between ischemic and bleeding risks is certainly recommendable before deciding the P2Y_12_ inhibition strategy in CAD patients.

### Limitations

We acknowledge several limitations of the present investigation, such as the open-label design and the relatively small sample size. Further, no pharmacokinetic or genetic (e.g., loss-of-function CYP2C19 alleles) assessments were done, which could have provided important insights on the mechanisms contributing to the differences observed in platelet reactivity between clopidogrel and ticagrelor. However, prior investigations in DM patients used a single platelet function assay to compare the PD effectiveness of ticagrelor vs. clopidogrel (10, 20), whereas four different assays were employed in the present study to evaluate the LD and MD effect, which yields a great consistency to the results obtained. Ultimately, the ticagrelor 90 mg b.i.d regiment is not routinely employed in long-term secondary prevention and our results cannot be extrapolated to the 60 mg b.i.d. dose of ticagrelor, which is approved in this scenario due to the results obtained in the PEGASUS–TIMI 54 trial.

## Conclusion

In Mediterranean DM patients with CCS, ticagrelor yields a more potent platelet inhibition than clopidogrel, which is detected promptly after the loading dose and is maintained after 1 week of treatment. This PD benefit results in significantly lower HPR rates with ticagrelor compared to clopidogrel both with the load and maintenance doses. Of note, ticagrelor HPR rates are almost negligible after 1 week of therapy. The present investigation is a valid confirmation of the consistent and favorable PD profile of ticagrelor among different high-risk subgroups, such as patients with DM.

## Data availability statement

The data analyzed in this study are not publicly available due to internal policy. Any requests can be directed to the corresponding author. Requests to access the datasets should be directed to JF, jlferreiro@bellvitgehospital.cat.

## Ethics statement

The studies involving human participants were reviewed and approved by CEIm Hospital Universitario de Bellvitge. The patients/participants provided their written informed consent to participate in this study.

## Author contributions

AM and JF contributed to the conception and design of the study, analysis and interpretation of data. AM acquired data for the work and drafted the manuscript, which was critically revised for important intellectual content by the other authors. All authors approved the final version submitted.
